# The Clinical Evolutions of Surveillance and Violence During Three Contemporary US Crises: Opioid Overdose, COVID-19, and Racial Reckoning

**DOI:** 10.1007/s11013-023-09842-4

**Published:** 2024-01-16

**Authors:** Kelly Ray Knight

**Affiliations:** Dept. of Humanities and Social Sciences, School of Medicine, University of California, San Francisco, USA

**Keywords:** Opioids, COVID-19, Clinical care, Racism, Surveillance

## Abstract

In 2020, three crises coalesced to transform the clinical care landscape of addiction medicine in the United States (US). The opioid overdose crisis (crisis #1), which had been contributing to excess US mortality for over two decades, worsened during the COVID-19 pandemic (crisis #2). The racial reckoning (crisis #3) spurred by the murder of George Floyd at the hands of police impacted clinical care, especially in safety net clinical settings where the majority of people targeted by police violence, and other forms of structural violence, receive healthcare to mend both physical and psychological wounds. Collectively, the three crises changed how providers and patients viewed their experiences of clinical surveillance and altered their relationships to the violence of US healthcare. Drawing from two different research studies conducted during the years preceding and during the COVID-19 pandemic (2017–2022) with low income, safety net patients at risk for opioid overdose and their care providers, I analyze the relationship between surveillance and violence in light of changes wrought by these three intersecting health and social crises. I suggest that shifting perceptions about surveillance and violence contributed to clinical care innovations that offer greater patient autonomy and transform critical components of addiction medicine care practice.

## Death Comes in Threes

As we lumbered into the third decade of the US opioid overdose crisis in early Spring and Summer of 2020, a second and third set of crises arose: the COVID-19 pandemic and the widespread social protest in response to racist US police violence. Collectively these crises disrupted the ground on which we tread, morphed the sight lines that we relied upon to see and understand, and produced on-going, numbing, excess mortality.

Human beings have a tendency, perhaps especially in times of crisis, to assume an additive effect of events as a rational explanatory practice. The logic of an additive, or cumulative, effect is that more must mean “more”, and often must also mean “worse”. This is commonly known as catastrophizing in the field of human psychology. Epidemiology, as quantification fetish, also directs us toward the additive. With both psychology and statistical thinking to guide us, it is natural to assume that the three simultaneous social/health crises (1) the opioid overdose crisis, (2) the crisis of the COVID-19 pandemic, and (3) the crisis of racist police violence that produced a racial reckoning in the form of widespread social protest, would cause overall health and wellbeing to worsen. And, in many ways, these three crises have acted synergistically to do just that. Overdose death increased significantly during the COVID-19 lock-downs, when people were more likely to use opioids alone and illicit drug supplies became increasingly unsafe (CDC, 2022). Substance use of all kinds also increased and became more chaotic for many people during the pandemic and in response to the on-going murder of Black-identified people in the United States without accountability or justice (Campbell & Valera, 2020; Grossman et al., 2020; Jordan et al., 2021; Wainwright et al., 2020). Anxiety, depression and suicidality significantly increased in 2020 and 2021, across all US age groups (Farooq et al., 2021; Kumar & Nayar, 2021). Those are statistical, well-documented facts that reflect the aggregated experiences of many people across the US. While critical to document those facts as one mode of monitoring population health (Krieger, 2020), it is important to attend to the nuance more specifically to understand the experiences people who lives and clinical care practices that exist at the intersections of these three crises.

Sam Quinones, a journalist who has written extensively on the US opioid crisis, recently made broad claims about how racist police violence, COVID-19, and the US opioid crisis might intersect, saying: “Each was about who in America could breathe and who could not. George Floyd’s final words were those, too, of the addict dying under the overpass, and the trucker expiring from COVID-19” (Quinones, 2021). This gloss may be appealing. Yet it is also an oversimplification, a desire for the singular, grand narrative that explains all by wrapping up profound structural differences, circumstances, and histories into a unified package. “Deaths of Despair” discourses produced by economists are equally at risk of losing the nuance, perhaps due to commitments to a plotline (Case & Deaton, 2015).

Considering opioids as ethnographic objects of concern can allow for generative new ways of thinking about the human, the chemical, and the institutional (Knight, 2017; Rochel De Camargo & Kapadia, 2022). I have often heard medical students that I teach say: “The opioid crisis is our AIDS” which speaks to the very real urgency and overwhelm that surrounds the morbidity and mortality associated with opioids, while also referencing the complex social and political challenges that travel along with and in the wake of the opioid overdose crisis. Paula Treichler (1999) famously provoked those of us working at the intersection of medical anthropology and public health by asking: “How do we have theory in an epidemic?” Talking about HIV/AIDS, she argued for the examination of the multiple modes of signification that surround health crises to reveal both the structural violence of institutional neglect and the on-going, entrenched invisibility of those made most at risk through stigmatization. All three of the crises described in this paper have been framed with the language of “epidemic” – the COVID-19 pandemic, “the opioid epidemic” and a US epidemic of racialized police violence – underscoring their insidious and dangerous characteristics, those which threaten the social body as well as the health and lives of individuals. Having theory in an epidemic articulates what feels both contagious and widespread about these intersecting crises, helping to identify fears and entrenchments, as well unexpected possibilities for positive transformation. It allows us to get concrete about the ways in which medical anthropological theory and ethnographic methods can help us make sense of the care practice changes we see emerging and understand their consequences (McNeil et al., 2022). This is particularly true when the violence that individuals experience within and from healthcare institutions becomes a stark realization of their experiences of intersectional structural stigma (Walters et al., 2023).

The carceral logics of the US War on Drugs shape the organization of clinical care and the contours clinical care experiences for patients who are at risk for opioid overdose. The linkages between surveillance and violence have been charted in scholarship describing the intertwined histories of the development of policing and medicine in Europe and the United States (Foucault, 1994; Fernández, 2023); the policies and practices that support racialized mass incarceration (Alexander, 2010) and family policing of people who use drugs (Roberts, 2022); and, the clinical care apparati that manage the physical and mental health consequences of substance use through the deployment of racial capital (Hansen, 2019; Hansen, Netherland, & Herzberg, 2023). Scholars Kaba and Ritchie (2022) have connected the actions of traditional policing with those of policing by medical professionals through their conceptualization of “soft police” – professionals who significantly impact the lives of low-income patients due to their ability to grant or withhold clinical treatments, benefits, and privileges. In this manner, surveillance and violence coexist and characterize care for people who use drugs in the broad array of clinical settings in the United States (Fernández, 2023).

I am interested in analyzing the processes and unexpected consequences that occur when care patterns that are historically shaped by surveillance and violence evolve in response to crises. Medical anthropologists have examined the role of crisis in the construction of clinical subjectivities and changing demands on the presentation of patienthood, for example in the context of geopolitical conflict and refugee asylum claims-making (Redfield, 2013; Tichtin, 2011). This work demonstrates how humanitarian crises can produce negative consequences that further patients’ vulnerability to harm and reinforce structural violence in the name of benevolent care. Here, I theorize about the potential for collateral gain, rather than collateral damage. The complex intersection of the opioid overdose crisis, COVID-19, and social reckoning related to racialized police terror created an opening for clinical care practices that had been previously undoable in the US safety net for methadone treatment delivery and chronic non-cancer care treatment. These crises created shifts in expertise in which new care configurations relied less on surveillance, and its inherent violence, and promoted a measure of patient autonomy. In this paper, I explore the liberatory potential that can emerge when intersecting crises make demands on clinical subjectivities of providers and transform the clinical care experiences of patients.

## Two Studies of Three Crises

This work draws on multiple years of home-based ethnography, interviews, and clinical observations between patients at risk for opioid overdose who received care in safety net clinical settings in the San Francisco Bay Area. The first study focused on patients with a history of substance use who have diagnoses of chronic non-cancer pain and their primary care providers who practice in primary care safety net settings. From this study, I describe how pain and opioid management of patients became reinterpreted through a novel clinical technology in this setting – telemedicine, in relationship to the three intersecting crises. The second study is focused on patients and providers in one Opiate Treatment Outpatient Program (OTOP) who experienced a radical reconfiguration of methadone take-home dose availability as a result of a need to depopulate clinics while not disrupting access to medications of opioid use disorder. Methodologies employed in both studies, which are described, at length, elsewhere (Cooke et al., 2023; Suen et al., 2022a), include semi-structured qualitative interviews with patients and providers; and, participant observations of clinical care and home environments. For the chronic non-cancer pain study, five primary care clinics were selected that served patients in outpatient hospital and community settings in three SF Bay Area counties. Provider participants were recruited via clinic staff meetings and email follow-up, and patients were recruited via provider referral. For the methadone care delivery study, the OTOP clinic was selected based on its implementation of changes in take-home dose policies at the beginning of the COVID-19 pandemic. Providers and staff were recruited via one clinic staff meeting and email follow-up. Methadone patients were recruited, using convenience sampling methods, at the outdoor methadone van during dosing hours, and through provider referral. Interviews for the chronic pain study focused provider and patients experiences with chronic pain management, opioid tapering, telehealth, patient-provider relationships, and systems-level facilitators and barrier to care. The methadone study interviews addressed provider and patients experiences with the implementation of the changes in methadone care delivery practices, including increased eligibility for take-home dosing. Interviews were transcribed verbatim and codebook develop and coding was conducted. For this analysis, the author analyzed codes related to telemedicine, care practices changes, COVID-19, methadone take-homes, surveillance, racism, social context, and social protest.

## Complex Social and Physical Etiologies of Multi-Morbidity

The group of patients in both studies were interpellated through clinical care settings as being at risk for opioid overdose. In the primary care setting study, all of the patients had a clinical diagnosis of chronic non-cancer pain (CNCP) and had been prescribed opioids. In the methadone clinic study, many patients also had pain diagnoses. Across both studies, many were using, or had used, opioids obtained both through prescription and outside of prescriptions, and other substances such as cannabis, alcohol, benzodiazepines, and stimulants. One of the often-misrecognized facts produced by silo-ing patient characteristics by singular diagnoses and “drug of choice” is that multiple morbidity and polysubstance use are normative (Ciccarone, 2021; John et al., 2018). The structural vulnerabilities we produce at the societal level produce increased risk for unequal burdens of chronic and infectious disease diagnoses, and shore up differential, and worse treatment outcomes once ill (Kreiger, 2004; Galea & Vlahov, 2002).

Much energy is expended in parsing patient populations into the categories of “chronic non-cancer pain patient” and “opioid use disorder patient” or less kindly, “legitimate pain patient” and “drug seeker” or even less kindly, “malingerer” and “addict.” Yet, the cumulative disease burden of patients is significant, consequential, and often anticipated by patients themselves. Many anthropologists, social scientists, and humanities scholars have aptly pointed us toward the exceptional nature of pain, and its treatment, in clinical settings (Scarry, 1985; Good et al., 1994; Greenhalgh, 2001; Crowley-Matoka, 2012; Wailoo, 2014; Buchbinder, 2015). In addition to this singular focus on pain, we must also attend to pain’s integration and entanglement, not only with addiction, but with overall poor health and extensive social trauma (Pryma, 2017; Tsai et al., 2019). The majority of patients in these two studies had *an average* of three chronic conditions *in addition* to chronic non-cancer pain and opioid use disorder - most commonly diabetes, hypertension, and chronic lung diseases, such as COPD.

Providers expressed concerns about healthcare settings being a source of risk for COVID infection and also concerns about the impact of poorly treated chronic health conditions and overdose risk for patients of not attending brick and mortar, face-to-face clinical care. In this way the management of the COVID-19 pandemic crisis and the opioid overdose crisis were put in active juxtaposition, demonstrated by the calculus of risk that providers in the two studies described assessing. One primary care provider said: “It is exactly the patients who are the sickest, who we want to be able to see in person [in primary care clinics], who we absolutely need to try and keep at home because if they get COVID, they will fair very poorly.” Another provider, from the methadone clinic, said: “I think with COVID we're trying to weigh the risk of coming in [to receive a methadone dose] for somebody who's got, [like] patients who've got really severe COPD and every time they get on a bus to come here they're at risk of getting COVID, and so having to come in every day [to receive a methadone dose] versus once a week dramatically increases their risk [for COVID-19 infection].”

The dual risks for COVID-19 infection and opioid overdose in the context of multiple morbidity shaped how providers experienced their decreased ability to surveille patients in traditional clinical settings as both telemedicine and methadone take-homes were implemented.

## Telemedicine in Primary Care Pain Management

When shelter in place took effect in the San Francisco Bay Area on March 17, 2020, all non-urgent medical visits moved to a telemedicine format. Defined as “the use of medical information exchange from one site to another via electronic communications to improve a patient’s clinical health status” (American Telemedicine Association, 2013), telemedicine has a history of demonstrated effectiveness for the management of multiple chronic conditions (Hanlon et al., 2017; Jayakody, 2016). Telemedicine’s effectiveness in safely increasing access for medications for opioid use disorder (e.g., buprenorphine and methadone) had been assessed prior to COVID-19, especially in more rural geographic locations in the US where transportation barriers are significant (Weintraub et al., 2018). However, the widespread use of telehealth for urban patient populations receiving care for chronic non-care pain in primary care settings net settings did not exist prior to the COVID-19 pandemic (Cooke et al., 2023; Mehtani et al., 2021). It was necessary to adjust payer requirements and preauthorization procedures to create this exception for patients during COVID, raising concerns about equity and sustainability for this new clinical modality (Mehrotra et al., 2020; Nouri et al., 2020).

In this study, we had already been working with providers and patients when COVID-19 necessitated care practices changes to telehealth, and so we remained engaged with patient participants and their providers as they navigated this new clinical terrain. Risk, safety, isolation and containment were all newly operationalized in the context of COVID-19. But ideas about risk and safety moved far beyond opioids, expanding to include isolation, anxiety, depression, and racial trauma. Many patients described increased isolation that exacerbated their pain during this period. One patient told us: “I don’t like [the shelter-in-place policy] at all. I’m not worth one penny. I’m gaining weight. I’m depressed. I’m almost paralyzed from not doing anything…It’s really messing with my psyche...It just makes me not want to do nothing. I just sit here… I don’t want to clean. I don’t want to do anything.”

As a result of report of increased, uncontrolled pain, many providers increased opioid pain medication doses for patients or discontinued opioid tapers that were actively in place prior to the pandemic. Providers recognized and worried about increased substance use and lack of social supports and stability. Many providers described patients returning to substance use after being abstinent prior to the pandemic or escalating use significantly in response to the fear and anxiety of potential COVID infection, shelter-in-place rules, and uncertainty about the future.

Prior to the co-arrival of COVID-19 and social protests against racist police violence in the Spring and Summer of 2020, this patient population were under intense surveillance and subject to multiple biopolitical interventions that managed their threats to personal and the public’s health resulting from their opioid use (Knight et al., 2017). During this telemedicine period, many patients who could not access community clinics or were experiencing acute symptoms, sought out emergency room visits for pain relief. One patient described the need to enlist her provider to manage the fact she was labeled “drug seeking” during an ED visit:

Before my surgery and I was in a lot of pain I’d go to the emergency they’d walk me out the door and set me on bench and said I was looking for drugs. And it offended me in a really big way. I felt like [they] degraded me. And so therefore [my doctor] has in my records and she shared [that] “This woman still [has] no drug history and she don’t even want to be on them [opioids]. And she’s been through a lot.”

Surveillance and violence traveled together producing vulnerability and codifying isolation during this period. Some pharmacies were vandalized and some chose to close during the street-based protests and city government curfews, disrupting access to opioids, and other chronic disease medications. One patient described the connection between her medication disruptions and the justified anger of those protesting George Floyd’s murder:

Participant (P): I got an emergency call for my [opioid] prescription to be picked up and they switched over to a pharmacy. Tomorrow is my pickup day and already I’ve been without my shit [opioids] for two days. I can’t take no more of this Motrin.

Interviewer (I): And tell me why did you change your pharmacy?

P: I didn’t change it, the people looted it and they were breaking up shit about this boy Floyd thing. They had to close it, they looted it, ran up in there and broke up shit. The pharmacy called me and made me aware that I wouldn’t be able to pick up my [opioid] medication there because of the looting and suggested that I put in a call to my doctor and have him switch my prescription that I’m supposed to pick up tomorrow to a pharmacy that’s fucking in a Walgreens and fuck it now I have to go to [city next door] to get it. I got no ride there either. And then believe it or not most of my friends are homeless and I probably could take the other motherfuckers in but I can’t stand my peace being disturbed, I’ve got enough shit going on in my life. But anyway I’m scared to ride with my damned friends with this Corona shit go on, like I say they sleep in their car, they leave shit in their car whatever they do…. Did you watch the whole video of the four officers on that boy [George Floyd]?

I:That’s just horrible.

P: I said did you see it?

I: I did. I didn’t see the whole nine minutes but I did see it.

P: You seen the part where the motherfucker [George Floyd] was begging for his life…I mean that would break anybody’s heart. Didn’t it break yours? It should have bothered you. The fact it’s being done to another human being without even considering. You see them motherfuckers [the police] took and drug his body...What kind of shit is that? But they [the protesters are] tired, the motherfuckers tired of everything because you motherfuckers [non-Black-identified people] ain’t going to do it if we don’t get to. That’s what’s happening. I understand it, hell yeah. But I sure wish them n----- would have left Walgreen’s alone. That’s the price I have to pay for being black to be honest with you.

Telemedicine served as a source of containment for patients who are multi-morbid and considered high-risk, and as an unlikely sight for connection. One provider described how conversations occurring via telemedicine visits had increased their awareness of the intersecting phenomena of pain, COVID-19, and the widespread social reckoning with racist police violence:

I was definitely nervous [starting telemedicine]. I still was used to [a previous clinic] where I could never get anybody on the phone so I wasn’t sure of how many folks we would reach. But yeah I’ve been very pleasantly surprised and it’s been a pretty smooth transition [to telemedicine]…I think one of the things we haven’t talked about in terms of just like burden of stress on patients is so many of our patients with multiple chronic conditions and chronic pain are African-American. And I think the added burden of kind of all of the racial justice reckoning that’s been going on and George Floyd’s death it really felt like a double whammy for some of the patients I talked to just you know just seeing you know Black people being killed by Corona virus, Black people being killed by the police really added an extra level of stress. And I think as a provider trying to figure how to, once I started hearing the patients [via telemedicine conversations] who were more comfortable bring it up, try to find ways to bring it up that didn’t feel re-traumatizing and I tried to prioritize that and I was just really struck by how hard hit folks have been.

Technological innovation and fear were both key actors in the new clinical care geographies that collapsed and conflated of boundaries between clinic and home during the three co-occurring crises. Providers feared patient opioid misuse, that patients’ social isolation would severely worsening mental health and substance use, that the social realities of structural violence would perpetually destabilize or destroy clinical gains, that the metrics of clinical progress might have lost relevance as their CNCP patient were constantly subject to the assaults of structural racism and poverty. Providers also expressed concerns about those patients who might be denied the benefits of telemedicine, due to lack of ability to pay for phone service or inability, due to living circumstances, to engage in confidential conversations about their health (e.g., patients living in crowded housing situations or experiencing homelessness). Patients feared increased social isolation, loss of access to opioid medications, further degradation through accusations of malingering, increased vulnerability to disease and death from COVID, and racial discrimination in healthcare settings (Cooke et al., 2023).

The innovation of telemedicine in the context of three co-occurring crises also created strange new openings for both patients and providers. Telemedicine visits enabled distancing, while also promoting some unique forms of connection. These connections were exclusively auditory. None of the clinics we were working with were able to use video-enabled telemedicine visits, explaining that the digital divide was too great to overcome. Overall, providers described feeling greater loss due to lack of the face-to-face interaction. Patients reflected that telemedicine decreased burden for them. They appreciated not having to travel to visits, see providers, or to be seen by them. Many patients felt “seen”, even though they were only heard.

Providers felt increased access, insight, and more disclosure mediated through a physical separations and temporal disruptions to care practice routines that COVID necessitated. As the clinic traveled through the airways, the clinicians saw things differently based on what they heard. With COVID-19, some previously routine surveillance practices, such as regular urine toxicology screening, were abandoned, producing new temporalities of autonomy and risk. One provider described telemedicine as “more patient-centered” in that patients could choose when to engage (e.g., whether or not they chose to answer their phones), but it also allowed for more direct requests for on-the-spot medication management from providers:

It is way more patient-centered which we’ve never been in [our system], which I think is really great. And I think you know there are some things you can say to people you know, “go get your bottles and read them to me” now [that] when they forget to bring them into the clinic and they can do that for you right there on the spot. I think some of those things are some of the really positive things.

## Methadone Take-Home Dose Liberalization

Methadone is a medication used to treat opioid use disorder, that has been shown to effective at reducing mortality and increasing stabilization because it helps to manage the cravings and discomfort associated with opioid withdrawal (National Academies of Sciences, Engineering, and Medicine, 2019). Once maintained on methadone, with withdrawal symptoms managed, methadone patients are often more able to pursue employment, education, and stable housing – all of which may have been compromised during more active opioid dependency (Mattick et al., 2009; Sun et al., 2015)

Methadone has a long history of being the most regulated and surveilled medication in the United States, for any chronic condition (McBournie et al., 2019). This history is intentionally linked to the geographic targeting of methadone outpatient clinic locations to inner-city, Black and Brown-identified communities, and the requirements for daily clinical attendance to receive needed methadone doses as a form of racialized social control exerted on people with opioid use disorders who are criminalized as a result of their drug dependency (Tabor, 1970; Hansen et al., 2013; Raz, 2017). Prior to COVID-19, patients had to meet multiple, strict criteria to be consider eligible for a “take-home” dose, which meant that the patient would be able to skip one or more days of daily attendance. The federal criteria to be eligible for take-home consideration included: “Absence of recent use of illicit or non-prescribed opioids, stimulants, benzodiazepines, alcohol, and other substances; Regular clinic attendance priori to take-home being offered; Absence of serious behavioral issues at clinic; Absence of known recent criminal activity; Stability of home environment (e.g. housing) and social relationships; Sufficient length of time in treatment (varies by clinic); Ability to safely store medications in a home environment; The rehabilitative benefit of decreasing frequency of clinic attendance outweighs potential risk of diversion” (SAMSHA, 2020a). Short of meeting these criteria, patients were tethered to daily attendance at the methadone clinic.

Any reading of these criteria brings the quick understanding that methadone clinics are highly regulated environments. What is also embedded in the list is the fact that methadone providers have a tremendous degree of autonomy in deciding which patients get access to the benefits of take-home dosing. As a result, methadone patients have historically struggled to advocate for themselves, especially if they have been deemed to be “problematic patients” or do not have therapeutic, trusting relationships with their methadone counselors, as those are the providers who often recommend or do not recommend take-home benefits (Simon et al., 2022; Suen et al., 2022a). To provide an example of the degree to which patients experienced surveillance and lack of control of their own opioid treatment trajectories, I offer a retelling of an experience of breached confidentiality of patient information that lost one study participant his employment and eventually his access to methadone treatment at a previous methadone clinic.

[M]y job had called my methadone counselor and I asked him do I dose here [at this clinic] and he was like yeah he doses here. But then all my counselor had to do was let them know [that I was a patient]. I told him, I said, “my job is calling they’re going to ask you do I dose here and do I dose here every day. All I want you to do is say, ‘yes’”. That’s all they need to know is I dose here every day. He went into [told my job], I come in late every day, 5 minutes before they close, every day. I never come in on time. I’m always late right before they close. He told him I had a dirty UA [urine toxicology screening] …he was basically telling them I’m using drugs, I’d be high at my job…So I got fired. And I went ballistic on them [the clinic]. And they was like, well you still gonna see your counselor? I said, “I’m not gonna see this man. I don’t trust him.” They broke my confidentiality. I don’t trust him. I need a new counselor. They said, “well you’re still gonna see him if you don’t see him you’re gonna have to get kicked off the clinic.” [I said] “Well, take me off the clinic.” And that’s just how it went.

This example demonstrates the difficulty patients face if they are not abiding my strict treatment guidelines and underscores the lack of agency many patients feel in relation to urine toxicology screenings and counseling requirements. The history of racialized methadone targeting and its intense clinical surveillance has been extensively examined by Helena Hansen and colleagues (Hansen, 2013; Hansen and Netherland, 2017), and their analyses have joined those of methadone patient researcher-activists who have called more liberalization of methadone treatment delivery practices for decades (Simon et al., 2022). Although many countries, such as Canada, Scotland, and Australia have expanded methadone availability to consumers through primary care and/or community pharmacy settings, little had changed in the US until the COVID-19 pandemic (Suen, 2022b).

In March of 2020, the Substance Abuse and Mental Health Services Administration led the federal mandate to permit blanket exceptions to methadone delivery to reduce the spread of COVID-19 by de-congregating methadone clinics and reducing one-to-one interactions between methadone providers and patients (SAMSHA, 2020b). These exceptions included allowing up to 14 days of methadone take-homes for “unstable” patients and 28 days of take-homes for “stable” patients. Methadone clinics had discretion in defining patients as “stable” or “unstable,” and these exemptions also provided other waivers related to urine toxicology monitoring and counseling (SAMSHA, 2020b). These changes represented an unprecedented liberalization of methadone care delivery in the United States and took place during a period of two crises, additional to COVID-19, that impacted the process of implementation and the assessment of outcomes.

The impact of racial reckoning that occurred concurrent to these sweeping changes was mediated through an understanding of racialized disparities in the history of methadone care and care delivery practices. It was with this understanding that providers at the methadone clinic attempted to attune themselves to equity as a framing lens through which to implement the new take-home policies. One provider stated:

And so by tracking very closely the race, ethnicity of the people who are on methadone… you want to balance the individual treatment planning and individual decisions about individual patients with the idea that they are part of the larger group. And we need to be fair about everything.

The majority of the patient population of the safety net methadone clinic relied on public transportation to travel to the clinic. Changes that arose from the COVID-19 pandemic and from the street protests associated with the racial reckoning the Summer of 2020, meant that buses ran less frequently, sometimes refused to stop and pick up patients, and sometimes stopped completely due to human traffic during the protests. As one patient said: “Whenever they have the [George Floyd] protests and stuff like that the buses would run like sporadically.”

For patients who experienced withdrawal if they were unable to access daily methadone, these disruptions perpetuated increased risk for accessing an unsafe, unregulated fentanyl supply, and thus increase risk for overdose. Getting access to more take-home doses meant access to a safe supply of opioids (methadone). Take-home doses also meant less bus rides, which meant less potential COVID exposure. As one patient stated: *“*Yeah, it’s a big difference. [Take-homes] helped me because at the beginning I go, ‘Thank god they gave me this, that I don’t have to come out, get on the bus,’ because at the beginning [of COVID] they weren't asking for masks [on the bus].” For patients with additional disabling chronic conditions that made travel difficult and increased their likelihood for serious health outcomes if COVID infected, access to take-homes significantly decreased their emotional and physical burden. One patient, who had a mobility disability that impacted their ability to walk long distances, described their struggle to access the clinic every day with an unreliable bus system and their desire to avoid close contact with others during the pandemic:

You know [my methadone counselor] seen me one day when I had to walk from my house to the clinic [because the bus did not pick me up] and the hills go up higher so she seen me and when I got there, and it was like two minutes before the clinic closed, and one day I missed because I couldn't get there so she said, “I’m going to start to get you take-homes so you won't have to come out here every day.” I stay at home more now since the COVID-19 hit because, it’s nothing out there for me anyway once I do the little errands that I have to run. But the clinic is extra for me, but that was something that I did seven days a week and I don’t have a car so the transportation is bad with the COVID-19, and it still is, believe it or not. […] Because it's a lot of mental patients get on the bus, they're going to see their psyches or whatever, and then, the homeless people. And who am I to talk about homeless people when I’ve been homeless before but I’ve always tried to take care of myself and it's no cure for the COVID-19. I don't want to go out like that, I just don't want to go out like that. If I did I probably would have stayed on drugs.

Even as the clinic strived toward an equity frame in determining access to expanded take-homes that was informed by the long history of racialized disparities in opioid treatment and surveillance, some patients’ honesty was not rewarded. The overdose risk associated with methadone and benzodiazepine co-use made it exceptional to the take-home expansion rule. While those who co-used stimulants and methadone were allowed take-homes under the COVID exception, those who reported or were discovered through urine screening to have used benzodiazepines were not allowed. One patient explained: “I can’t get take-homes because I got a dirty [a urine toxicology screen showing a positive result for benzodiazepines]. So it is what it is, I feel like these are liquid handcuffs.” Those patients who were suspected of selling their medications were also ineligible, as were those patients experiencing unsheltered homelessness because of the assumption that they not safely store methadone, to prevent its theft or misuse. While many patients benefitted, a significant minority of patients were left behind as a result of substance use, mental health, and poverty.

The change in the regulations that liberalized access to take-homes during COVID-19, spurred concrete conversations in which patients described reimagining methadone care delivery and themselves as patients. Patients described a desire to be able to dose methadone in ways that work best for them, rather than being bound to clinical routines and schedules. One participant described wanting a methadone prescription and using that prescription to guide their own dosing regime, stating: “If I had, if I had my choice I would just contact the doctor and have them give me pills [prescription methadone]. And then I could taper my own self off.” Providers also endorsed their reimaging of methadone care delivery brought about through the expansion of the availability of take-home doses. One provider described the policy producing more “honesty” among patients and more possibility to negotiate access to take-homes even in the context of continued substance use – a possibility that was foreclosed priority to COVID.

I think it's just been a lot more honesty because clients know that we have the ability to give them take-homes for other reasons, like it's just too burdensome for them to get to the clinic five days a week, perhaps they're older and they have mobility issues, like who knows what, but they know that they don't necessarily have to meet the same criteria, and so like I’ve had conversations with some clients that were very honest in the sense of like, ‘I’m still using, it's only a couple times a week, it's this much and it's usually at night, but like that doesn't change the fact, like that's what I was doing before but it's really difficult for me to get to the clinic.’

Another provider’s subjectivity was equally shifted as they could imagine a future in which take-home dose liberalization continued:

I’d be really interested in seeing if, if you’ve got all these patients and we haven't had an increase in mortality or bad outcomes, however you define those, maybe take-home rules don’t need to be as stringent. We have a bunch of patients who have been using stimulants for a long time who seem to have done really a fine job in terms of managing take-homes and like maybe they should have take-homes.

## Toward a Liberatory Addiction Medicine?

The clinical settings in both studies experienced care practices changes (telemedicine and increased take-home methadone doses) that directly resulted from efforts to reduce transmission of the SAR-CoV2 virus at the onset of the COVID-19 pandemic beginning in March, 2020. However, the reflections of both patients and providers underscore how the implementation of the care practice changes reflected not only efforts to mediate infection during the pandemic, but also changing clinical subjectivities. These care practices changes were webbed into larger conversations about patient autonomy and clinical control as consideration of the relationship between surveillance and violence, for this patient population was, and is, highly racialized and shaped by discrimination against people with opioid use disorders. The enactment of intersectional stigma, stemming from racialized and under-resourced care delivery structures and experienced by patients with dually marginalized social positions as people of color and people who use drugs, was an omnipresent reality in primary care and methadone delivery (Castellanos et al., 2023; Cooke et al., 2023; Walters et al., 2023). However, care delivery changes that emerged contemporaneous to the triple crisis of COVID-19, opioid overdose, and racial reckoning opened up spaces in which stigma might be mediated through greater patient autonomy.

While extensive clinician anxiety about patient opioid safety and clinician dissatisfaction with pain management practices and tools existed prior to the emergence of COVID-19, once COVID arrived telemedicine was seen as the only clinical tool that could keep multi-morbid patients out of healthcare settings, while also still keeping them tethered to care. Both patients and providers experienced differentiated forms of transformation, as patients experienced less burden and providers came to listen differently to patients’ health concerns and attend differently to their social and home lives. In the case of methadone care delivery, patients were transformed from clinical captives, those forced to dose at the clinic and endure urine toxicology screenings and mandatory counseling sessions, into a new form of methadone consumer, those who could self-regulate their relationship to a medication. For the methadone providers, they recognized the regulatory structures that they were enabling were less necessary for patient protection (e.g., overdose risk mediation) and more in place as artifacts of historically racialized clinical hierarchies. And even while attending to equity informed by the current climate of racial reckoning, not everyone benefitted equally from the liberalized policies, especially those who used certain substances or remained unhoused (Suen et al, 2022b; Wyatt et al., 2022).

Jarret Zigon (2019) suggests an “anthropology of potentiality” that might help unseat the stagnation of oppressive clinical structures and destabilize the false temporality of crisis as the only instigator of sustainable and desired change. In exploring “drug user politics” as drug users construct and enact them, Zigon argues that “a key aim…was the need to change the conditions, or what we might called the *onto-interpretative matrix,* through which the drug war and its consequence are understood” (page 19). The onto-interpretative matrix in this case might include clinical diagnoses; federal regulatory bodies and pandemic response exceptions; language about racism and racial violence; conversations instead of, or in addition to, urine toxicology screenings; geographies of home and clinic; an historical lens pointed to the safety net and the patients and providers that inhabit them; and, ethnographic engagement. It is the undoing, or unthinking, of addiction medicine as a paradigm and praxis of surveillance - that may lead us in the direction of what Zigon (2019) calls “a global political movement that is attempting to build new worlds, to create new beginnings, and to bring about an otherwise” (page 26). The care practices changes experienced in addiction medicine during the three co-occurring crises produced an otherwise, and deeply altered what patient and providers thought was possible in terms of care delivery and treatment.

A recent re-engagement of *P*e*dagogy of the Oppressed* by Andre Gomes (2022) examines Freire’s critique of oppression through a drug policy lens. The oppressive structures here are the policies – namely drug prohibition – and the oppressors are policy makers who implement the laws and regulations that contribute to drug-related harms (e.g., Drug War policies that lead to mass incarceration). Extending this example, it is productive to examine how clinical policies in relation to the care practices of addiction medicine can generate oppressive surveillance in the name of safety (from opioid overdose), while not improving health. Walters (2023) argues that intersectional stigma functions as a fundamental cause of health disparities among racialized individuals who use drugs precisely because new routes of administration for policies that produce structural oppression are forged when policies that alleviate structural oppression gain ground. Thus, intersecting stigma can remain impactful, even in light of policy reforms.

The COVID-19 pandemic was a singular crisis that enabled both telehealth and take-home dosing in two clinical settings in which patients were obligated to undergo frequent and intensive clinical management as a result of their being prescribed legal opioids (opioid pain medications and methadone). The additional crisis of racial reckoning impacted the subjectivity of both patients and providers in clinical settings in which the majority of providers were white-identified and the majority of patients were Black-identified. The violence of racism intersected with the structural violence of poverty and manifested in and on the bodies of patients who experienced the burdens of multiple chronic conditions, disability, and poor health. The three crises converged to change the practice of medicine, changing the ways patients and providers saw themselves as clinical and social actors in dynamic and volatile social and clinical settings. The care practice changes also produced unexpected outcomes and new ways of experiencing complex chronic disease, medical surveillance, and structural suffering. These crisis-driven transformations created a concrete imaginary for more liberatory forms of addiction medicine which now include ongoing calls for reform to methadone treatment delivery and advocacy for the maintenance of forms of telemedicine for the treatment of chronic non-cancer pain and opioid use disorder, in recognition of the need to attend to social and structural circumstances that shape care (Cerda, Bennett, & Knight, 2023; Dasgupta et al., 2018; Simon et al., 2022). The question remains whether and how the collateral gains that these three crises have wrought will manifest into sustainable reforms in US healthcare for people at risk for opioid overdose, establishing clinical practices that delink surveillance, at its inherent violence, with care.
